# Supplementary material for: Avian Cell Line DuckCelt®-T17 Is an Efficient Production System for Live-Attenuated Human Metapneumovirus Vaccine Candidate Metavac®
Source: Vaccines (Basel). 2021 Oct 16;9(10):1190. doi: 10.3390/vaccines9101190 (PMC8540687; doi:10.3390/vaccines9101190)
Supplement: Supplementary file 1 [file vaccines-09-01190-s001.zip › vaccines-1399103-supplementary.pdf]

Supplementary Table S1 – Induction of neutralizing antibodies by Metavac® viruses in mice

Prime infection	Inoculum (log_{10} $TCID_{50}$)	Boost 30-day post-prime	Inoculum (log_{10} $TCID_{50}$)	Reciprocal neutralization titer (n=3)[a] Against the rC-85473-GFP (LLC) virus	
				29 days post-prime	21 days post-boost
Mock	-	rC-85473-GFP	5.7	< 5	10
		Mock	-	< 5	<5
rC-85473-GFP	5.7	Metavac® LLC	5.7	5	>160
		Metavac® T17	5.7	5	>160
		Mock	-	5	5

[a]Three pools of sera from two mice were tested for neutralization against the rC-85473-GFP (LLC) virus, resulting in three biological replicates per group. One day before prime infection, the naïve status of mice was confirmed by a microneutralization assay from a pool of sera.
